# Supplementary material for: Disrupting Bordetella Immunosuppression Reveals a Role for Eosinophils in Coordinating the Adaptive Immune Response in the Respiratory Tract
Source: Microorganisms. 2020 Nov 17;8(11):1808. doi: 10.3390/microorganisms8111808 (PMC7698589; doi:10.3390/microorganisms8111808)
Supplement: Supplementary file 1 [file microorganisms-08-01808-s001.pdf]

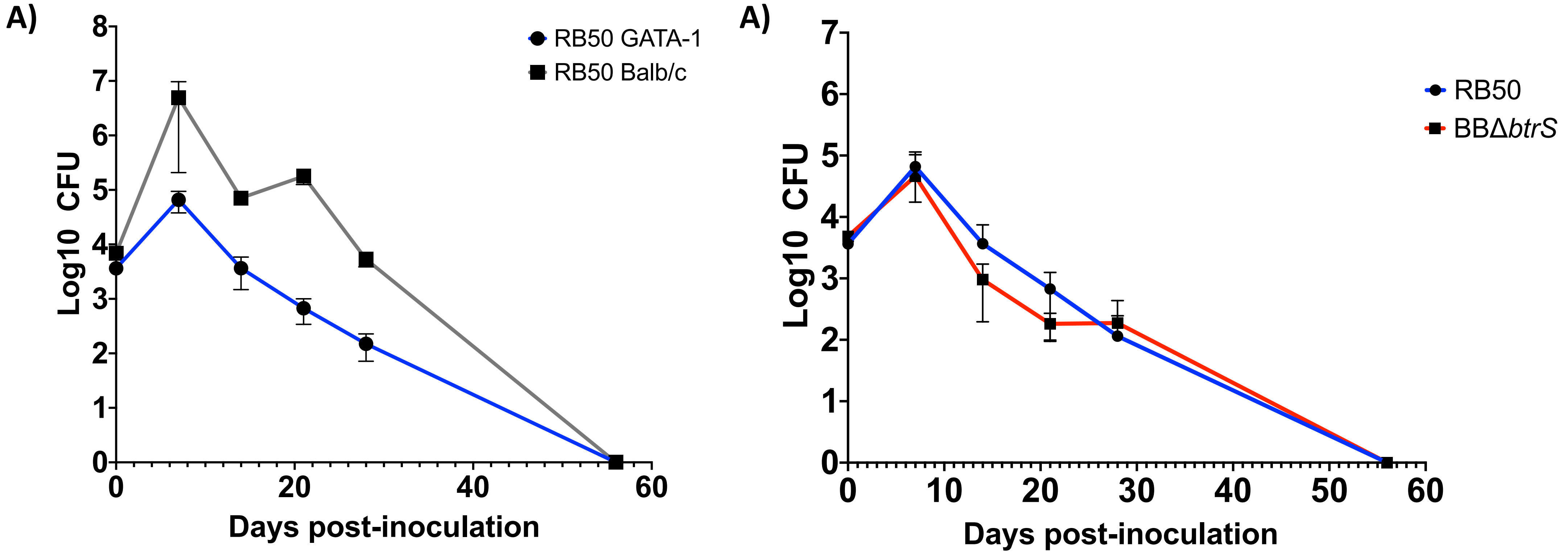

Figure S1: Colonization levels in the lungs of Balb/c and ΔdblGATA-1 mice following infection with RB50 and BBΔ*btrS*. Mice were intranasally inoculated with PBS containing $5x10^{5}$ CFU of RB50 (blue in GATA-1 and grey in Balb/c) or BBΔ*btrS* (red). Mice were euthanized at different times to enumerate colonization levels in the lungs. Lungs of Balb/c (grey) and ΔdblGATA-1 (blue) revealed similar levels of colonization when challenged with RB50 (A). ΔdblGATA-1 challenged with RB50 (blue) or BBΔ*btrS* (red) presented similar colonization dynamics (B). N= 6-10 mice per condition and time point
